# Automated delineation of stroke lesions using brain CT images

**DOI:** 10.1016/j.nicl.2014.03.009

**Published:** 2014-03-21

**Authors:** Céline R. Gillebert, Glyn W. Humphreys, Dante Mantini

**Affiliations:** a Department of Experimental Psychology, University of Oxford, Oxford, UK; b Department of Health Sciences and Technology, ETH Zürich, Switzerland

**Keywords:** Stroke, Computerized tomography, Lesion segmentation, Medical imaging, Software tool

## Abstract

Computed tomographic (CT) images are widely used for the identification of abnormal brain tissue following infarct and hemorrhage in stroke. Manual lesion delineation is currently the standard approach, but is both time-consuming and operator-dependent. To address these issues, we present a method that can automatically delineate infarct and hemorrhage in stroke CT images. The key elements of this method are the accurate normalization of CT images from stroke patients into template space and the subsequent voxelwise comparison with a group of control CT images for defining areas with hypo- or hyper-intense signals. Our validation, using simulated and actual lesions, shows that our approach is effective in reconstructing lesions resulting from both infarct and hemorrhage and yields lesion maps spatially consistent with those produced manually by expert operators. A limitation is that, relative to manual delineation, there is reduced sensitivity of the automated method in regions close to the ventricles and the brain contours. However, the automated method presents a number of benefits in terms of offering significant time savings and the elimination of the inter-operator differences inherent to manual tracing approaches. These factors are relevant for the creation of large-scale lesion databases for neuropsychological research. The automated delineation of stroke lesions from CT scans may also enable longitudinal studies to quantify changes in damaged tissue in an objective and reproducible manner.

## Highlights

• We present an automated method to detect brain lesions in stroke CT images.
• The method accurately warps CT images of patients (and controls) to template space.
• It detects lesion areas as those having abnormal signal compared to control images.
• It yields lesion maps spatially consistent with those produced by expert operators.
• Large-scale lesion databases for neuropsychological research can be created.

## 1 Introduction

Neuropsychological investigation of individuals suffering from stroke is widely used in cognitive neuroscience to advance our understanding of brain function. The analysis of correlations between impaired behavior and physical brain damage has provided considerable insight into how function depends upon structure (Fellows et al., 2005; Geva et al., 2011; Rorden and Karnath, 2004; Utz et al., 2013). In this regard, an exact delineation of stroke lesions constitutes a crucial step for structure/function studies in the field of neuropsychology (Seghier et al., 2008; Stamatakis and Tyler, 2005; Wilke et al., 2011). The typology of stroke can be broadly classified into two categories: 1) hemorrhagic stroke due to rupture of a blood vessel, and 2) ischemic stroke or infarct due to an interruption of blood supply. Of these, ischemic stroke occurs more often, and it is also possible for the two types of stroke to co-occur (Berger et al., 2001). Computed tomography (CT) and magnetic resonance imaging (MRI) are the two modalities regularly used for stroke lesion mapping. Though it is not unusual for MR anatomical images (usually T1- and T2-weighted images) to be acquired in stroke patients participating in clinical research protocols, CT is the preferred procedure in the acute stroke unit, typically offering the advantages of speed, cost, and reduced exclusion criteria relative to MR imaging (Rorden et al., 2012). On the other hand, MR imaging is earlier at detecting ischemic stroke, and if available, is therefore performed in many cases with a negative CT scan. In CT images, a hemorrhage appears as a bright region (hyper-intense) displaying sharp contrast against its surroundings. Conversely, an ischemic stroke appears as a dark region (hypo-intense), with the contrast relative to its surround depending on the time elapsed since the stroke occurred.

The standard method for lesion identification is currently the manual delineation of abnormal brain tissue by trained professionals (Fiez et al., 2000); however, this method has a number of disadvantages (Ashton et al., 2003). Other than being very time-consuming, the manual method produces variability across operators because there is often no clear cutoff between lesioned and non-lesioned tissues, particularly at the borders of the brain and around the ventricles. Furthermore, in chronic stroke patients, manual delineation typically does not detect inevitable stroke-induced degeneration that takes place outside the lesion, even though this degeneration can contribute to the clinical deficits in a patient. The automated detection of hypo- or hyper-intense regions, in combination with manual editing, can significantly reduce delineation times, but results still remain operator-dependent (Wilke et al., 2011). More recently, fully-automated approaches have been suggested, with the aim of removing inter-subject variability in brain delineation procedures and allowing for the analysis of large CT datasets (Rekik et al., 2012). While several algorithms have been developed for lesion segmentation in MR images (Seghier et al., 2008; Stamatakis and Tyler, 2005; Wilke et al., 2011), only a few methods have been proposed for CT scans of stroke lesions. Most existing work with CT images has been directed towards the detection of hemorrhagic strokes. Since hemorrhagic stroke appears brighter than normal tissue, unsupervised “fuzzy clustering” techniques have been proposed for detecting hemorrhagic candidates, followed by expert-based system labeling and morphological operations to distinguish the true lesioned regions from image artifacts (Chan, 2007; Cosic and Loncaric, 1997; Liu et al., 2008). In comparison to image-processing techniques related to hemorrhagic stroke, considerably less attention has been given to the detection of ischemic stroke, due to its more challenging nature. To address this issue, seeded region-growing algorithms have been used to segment a CT image into a set of regions having uniform intensities. Subsequently, features of these regions, such as brightness, extent, texture and relative position with respect to an axis of symmetry, have been given as input to a rule-based expert system to detect the region of the stroke (Matesin et al., 2001; Usinskas et al., 2004). The main disadvantage of this approach is that the boundaries of the stroke region may not be clearly defined by seeded region-growing algorithms. Moreover, to date, only a single study has addressed the problem of detecting both hemorrhagic and ischemic strokes in a given CT volume (Chawla et al., 2009). In this study, lesioned tissue was identified by comparing image intensities in the two hemispheres, under the assumption that an abnormal region in one hemisphere will have a significantly different intensity compared to the other hemisphere. This approach is imperfect, however, in that it cannot detect symmetrical abnormalities occurring on both sides with respect to the brain midline. Clinical data indicate that symmetrical abnormalities are a possibility, although the number of such cases is limited (Ganesan et al., 1999).

An alternative approach for the detection of hemorrhagic and ischemic strokes utilizes a comparison of CT image intensity from a single patient with a group of images from control subjects, in order to define outlier voxels. This approach has been successfully used, with several variants, in the analysis of MR images (Stamatakis and Tyler, 2005), but has never been applied to CT images. Significantly, this approach requires an accurate spatial registration of the individual brains to the same template image, and the first high-resolution CT template was published only recently (Rorden et al., 2012). It is worth mentioning, however, that the availability of a high-quality template does not in itself ensure a successful spatial normalization (Ripolles et al., 2012). Classical algorithms use affine and/or nonlinear warping to match a single brain image to the template. Problems can therefore occur when the brain to be normalized contains atypical areas of hypo- or hyper-intensity. In this case, more sophisticated spatial registration techniques, such as cost-function masking or surface-based registration techniques (Klein et al., 2010), can prevent the presence of a stroke from negatively affecting bias normalization in terms of an over- or under-fitting of the lesion area (Ripolles et al., 2012).

In this study, we develop a method that accurately normalises CT images from stroke patients to template space and applies subsequent voxelwise comparison with a group of control CT images in order to define areas with hypo- or hyper-intense signals. We demonstrate the validity and effectiveness of our approach by using simulated lesions superimposed on lesion-free brain CT images, as well as CT images collected from stroke patients. We anticipate that our method will permit automatic delineation of stroke lesions, and, in so doing, can provide a rapid and efficient tool for both research and clinical application.

## 2 Methods

### 2.1 Method description

We have developed a fully automated tool permitting preprocessing of brain CT images from healthy subjects and patients, as well as statistical analyses of lesion mapping. We validated the method using the CT image database collected for the Birmingham University Cognitive Screen (BUCS) project (*http://www.bucs.bham.ac.uk*), and obtained from stroke units across the West Midlands area of the United Kingdom. The CT data were expressed in Hounsfield units, a quantitative scale for describing radiodensity (Hounsfield, 1980) ranging from −1000 (value outside the head) to 1000 (value corresponding to the bone).

#### 2.1.1 CT image preprocessing

**Fig. 1:**
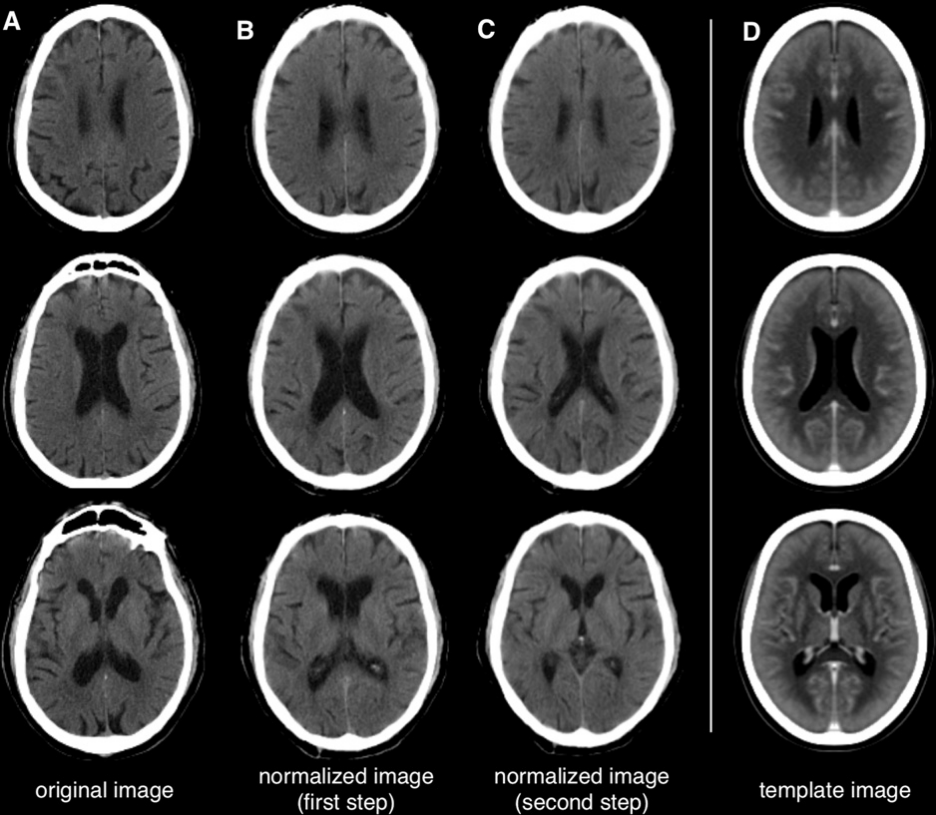
Spatial normalization of CT images to MNI space. A representative brain CT image, shown in three axial views. Section (A) is warped to MNI space in two steps (B–C), using a template CT image (D) as reference image. Note that the first normalization step registers the brain contour to the template, whereas the second step primarily registers periventricular regions.

CT image preprocessing of both patient and control data was performed using SPM8 (the Wellcome Trust Centre for Neuroimaging, London, UK) and in-house software written in MATLAB (The MathWorks, Natick, MA, USA). The same procedure was used for CT data collected from both patients and controls. First, we removed the irrelevant signals from the neck and sides of the head by applying a threshold-based clustering at 0.1% maximum intensity (Batenburg and Sijbers, 2009) and we spatially aligned the resulting CT image to the template image using the coregistration tool in SPM8. Subsequently, CT image intensity was transformed using an invertible formula to emphasize the image contrast between cerebrospinal fluid (CSF) and parenchyma, as proposed by Rorden et al. (2012). To implement this conversion, values from −1000 to −100 were rescaled to 0–900 by adding +1000, values from −99 to 100 were linearly scaled to the range 911–3100, and values where i > 100 were assigned the value [i + 3000] (Fig. 1A). We then warped the CT image to MNI space using the CT template (Rorden et al., 2012) (Fig. 1D), following a two-step approach. The first step involved the use of the SPM8 normalization function to calculate and apply a 12-parameter affine transformation that maximized the alignment to the template (Fig. 1B). We then calculated the distribution of all image intensities, providing the basis upon which the ventricles (signal < mean − 2 × standard deviation) and brain contours (signal > mean + 2 × standard deviation) were segmented (Volkau et al., 2010). Applying the ventricle and brain masks to the CT image produced a skull-stripped image that did not contain CSF voxels but did include lesioned voxels (Supplementary Fig. 1). Note that the invertible formula reduced the contrast between the lesioned and non-lesioned areas. This permitted the use of the same processing pipeline for both stroke and control scans because the normalization procedure was not substantially affected by the high contrast that typically characterizes a lesion. The skull-stripped image was then used for the second step of the normalization procedure. Specifically, a spatial deformation was calculated from the skull-stripped, scaled CT image to a skull-stripped scaled version of the template, and applied to the unmasked CT image (Fig. 1C). The resulting normalized image was next resliced at 1 mm isotropic resolution using a large bounding box that included both the cortex and the cerebellum (MNI-XYZ: min; max = [−90 −126 −82; 90 90,108]). In a final pre-processing step, the normalized CT image was smoothed with SPM8 using a Gaussian filter to accommodate the assumption of random field theory used in the statistical analysis (Salmond et al., 2002; Stamatakis and Tyler, 2005). The effects of different smoothing filters were examined in the study.

#### 2.1.2 Statistical analysis for lesion delineation

The lesion of each stroke patient was automatically identified using a voxel-based outlier detection procedure based on the Crawford–Howell parametric *t*-test for case–control comparisons (Crawford et al., 2009; Crawford and Howell, 1998). The result of this test can be expressed in terms of a *t*-score, defined as follows:

(1) $$\;t\;\text{=}\frac{x-\frac{1}{\text{n}}\sum_{\text{i=1}}^{\text{n}}c_{\text{i}}}{\sqrt{\frac{\text{n}+1}{\text{n}\left(\text{n}-1\right)}\sum_{\text{i=1}}^{\text{n}}{\left(c_{\text{i}}-\frac{1}{\text{n}}\sum_{\text{i=1}}^{\text{n}}c_{\text{i}}\right)}^{2}}}\;$$

where *x* is the value for the individual patient and *c*_i_ is the value for the i-th control subject, and n is the number of control subjects.

**Fig. 2:**
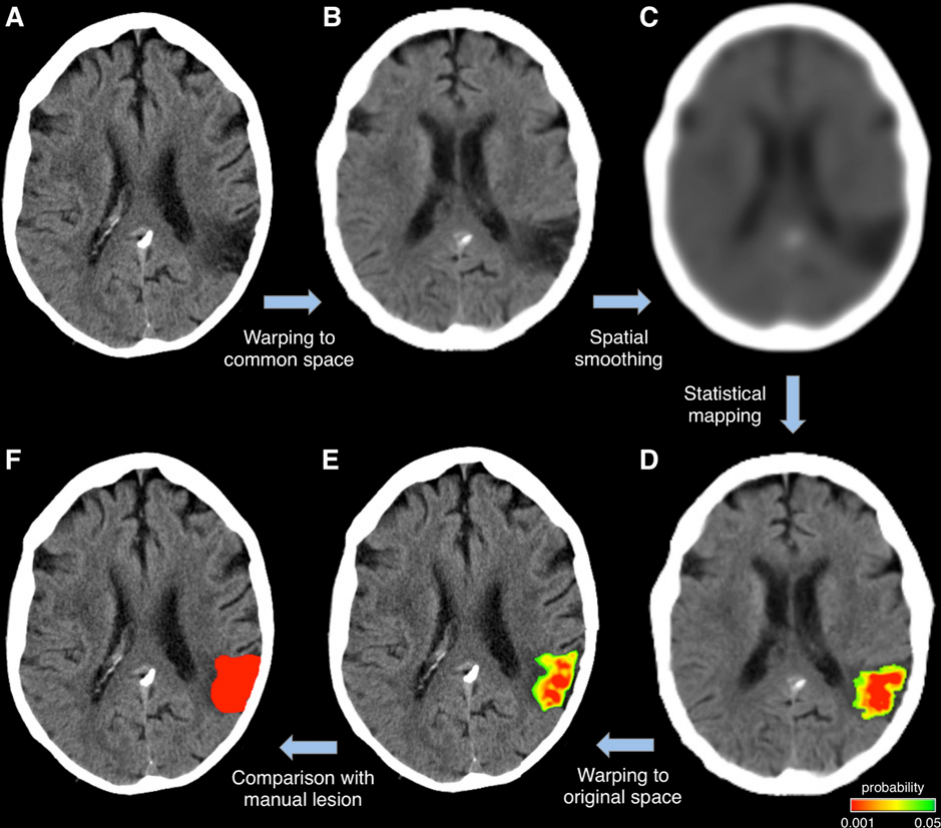
Classification of damaged tissue in stroke CTs. A representative stroke CT scan (A) is normalized to MNI space (B) and spatially smoothed (C). Next, the resulting image is compared to a group of control CTs by means of the Crawford–Howell *t*-test. The resulting *t*-score map is converted to a probability map, which is then overlaid onto the image itself (D). By thresholding this probability map at a given significance level, the lesioned regions can be delineated. The lesion map in MNI space can be transformed back to individual subject space (E), so that it can be compared with a lesion map manually delineated by an operator (F) on the original CT image.

Using the Crawford–Howell *t*-test, an outlier *t*-score map was generated that coded the degree of abnormality of each voxel intensity, based on the comparison to the normal range from control scans (Fig. 2). The outlier map generally contained both positive and negative values. By thresholding the *t*-score map at a given significance level, we obtained a lesion map that contained values of −1, 0 or 1. A value of −1 coded voxels with significantly lower intensities than normal and that most likely related to the presence of ischemia; conversely, a value of 1 coded voxels with significantly higher intensities than normal and that most likely related to the presence of hemorrhage. The lesion map in MNI space was also converted to the original CT space (before co-registration to the template) by inverting the spatial transformations used to move from individual to MNI space. This allowed a direct comparison with manual classification conducted on the original CT scan.

### 2.2 Method assessment

#### 2.2.1 CT data acquisition

CT scans were acquired as a part of their routine clinical assessment following hospital admission for stroke in eight stroke units across the West Midlands area (United Kingdom). All participants provided written informed consent to their inclusion in the BUCS project in agreement with ethics protocols approved by the National Research Ethics Service: Essex 1 Ethics Committee. The CT data were collected using the following scanners: Siemens Sensation 16 and GE Medical System LightSpeed 16 and LightSpeed Plus. The images covered the whole brain with an in-plane resolution of 0.5 × 0.5 mm^2^ and a slice thickness varying between 4 and 5 mm. A CT database of more than 500 patients with acute/subacute stroke was available. Acute and subacute strokes were defined respectively as strokes of less than one week and between one week and one month after onset. Clinical and demographic data were obtained from the patients' clinical files. Each patient completed a battery of neuropsychological tests for attention and executive functions, language, memory, and motor planning (Humphreys et al., 2011), in most of the cases within one week, and always within one month after CT scanning. We excluded CT scans where a shunt was visible or where the field of view of the scan did not completely encompass the head (*n* = 127). We classified the remaining 458 patients with valid CT images as having either a hemorrhagic or ischemic stroke and either a focal or extended lesion. For each of these four groups, we randomly selected six patients to assess the performance of our automated method with regard to stroke CT data as compared to manual lesion delineation (see Table 1 for detailed patient information). In addition, we selected 77 age-matched patients (Table 1) with no visible lesions on CT scans, who were used as controls. The neurological deficits in these patients were later found to be a consequence of metabolic abnormalities rather than stroke.

**Table 1:** Demographic and clinical information about selected stroke patients.The patients are divided into the following four groups: hemorrhagic focal stroke, hemorrhagic extended stroke, ischemic focal stroke and ischemic extended stroke. For each patient, the gender, the age, the time elapsed between the stroke and the CT scan, the type of the stroke, the main anatomical structure damaged and the lesion size are shown. Lesion location and size were obtained from the lesion delineation performed by an expert operator.

	Gender	Age (year)	Time to stroke (day)	Type of stroke	Lesion location	Lesion size (cm^3^)
Group 1						
P2059	F	72	0	Hemorrhage	Left superior temporal sulcus	2.1
P2065	M	65	0	Hemorrhage	Right superior temporal sulcus	9.1
P2173	M	81	0	Hemorrhage	Right basal ganglia	7.9
P2213	F	78	4	Hemorrhage	Right pulvinar	2.0
P2287	M	77	2	Hemorrhage	Left cerebellum	3.9
P2492	M	75	1	Hemorrhage	Left central sulcus	3.1
Group 2						
P2025	F	83	4	Hemorrhage	Left parietal	31.2
P2154	F	84	4	Hemorrhage	Left parietal	52.4
P2206	F	53	1	Hemorrhage	Right basal ganglia	14.9
P2374	M	57	2	Hemorrhage	Left putamen, left thalamus	12.6
P2474	M	61	3	Hemorrhage	Right cerebellum	16.8
P2713	F	73	0	Hemorrhage	Left insula, left thalamus	22.8
Group 3						
P2058	M	70	1	Ischemia	Left inferior frontal gyrus	3.2
P2064	F	79	2	Ischemia	Right occipital	2.9
P2066	F	83	0	Ischemia	Right dorsolateral prefrontal	9.0
P2091	M	74	2	Ischemia	Right basal ganglia	3.3
P2554	M	69	1	Ischemia	Right insula	8.4
P2624	F	57	1	Ischemia	Left basal ganglia	1.9
Group 4						
P2008	F	81	1	Ischemia	Left occipital, bilateral cerebellum	23.0
P2069	M	60	4	Ischemia	Left premotor	43.6
P2077	F	84	1	Ischemia	Right basal ganglia	12.4
P2342	F	75	4	Ischemia	Left frontal, right parietal	27.8
P2670	M	67	1	Ischemia	Right occipital	19.6
P2758	M	82	2	Ischemia	Right orbitofrontal	50.0

#### 2.2.2 Simulated lesion analysis

To assess the performance of our lesion delineation method, we created CT images containing artificial brain lesions with controlled incremental variations in size and signal. Spherical binary masks were defined in the same space of 5 images extracted from the set of control CT scans (5 male subjects, aged 46–75 years), and were respectively centered on the following MNI coordinates: [32, 12, −4], [15, −43, 9], [3, 55, −5], [−48, 6, 0], and [−21, 6, −13]. For each CT scan, we generated spherical lesions with different radii: 7, 10, 15 and 22 mm (i.e. size = 1.4 cm^3^, 4.2 cm^3^, 14.1 cm^3^ and 44.6 cm^3^, respectively). These lesions were then superimposed on the corresponding CT image, with intensities of −60%, −40%, −20%, + 20%, + 40%, and + 60% compared to the average CT signal in the brain. This range was chosen to realistically simulate the signal decrease/increase associated with ischemic/hemorrhagic stroke lesions (Supplementary Fig. 2). The resulting 120 (5 scans × 4 lesion sizes × 6 signal intensity changes) simulated CT images were subjected to the automated lesion delineation method, and the estimated lesion was then compared with ground-truth binary masks. Normal, CT intensity ranges, to be given as input to the automated method, were obtained using CT data from a group of 72 subjects without stroke (31 females, 69 ± 12 years old), which did not include the 5 subjects used to create the simulated lesions. We quantified performance in lesion delineation by measuring Dice's similarity index (DSI) (Zou et al., 2004). This is a measure of the similarity between the reference image (*X*) and the estimated image (*Y*) and is calculated as:

(2) $$\text{DSI}=\frac{2\left|X\cap Y\right|}{\left(\left|X\right|+\left|Y\right|\right)}$$

where |*X* ∩ *Y*| indicates the number of voxels that are common to *X* and *Y*, whereas |*X*| and |*Y*| are the number of voxels that are in *X* and *Y*, respectively. DSI ranges from 0 to 1; it is equal to 0 when there is no overlap between the estimated and reference images and is equal to 1 when they perfectly overlap. Accordingly, larger values indicate better lesion delineation performances (Zou et al., 2004). By using the DSI, we quantified the performance of our method using different smoothing levels (from 2 to 15 mm full width half maximum, FWHM) and significance (*t*-score) thresholds. In this manner, we identified the configuration of parameters that maximized detection performance for our automated method. These settings were then used for the analysis of the stroke CT scans.

#### 2.2.3 Analysis on experimental data

Initially, we assessed the reliability of our method by analyzing the five control scans that were used for the creation of the simulated lesions. The expert analysis detected no lesions in these images which were therefore used to quantify the detection of false positives by automated lesion delineation. We subsequently applied our lesion delineation method to stroke CT images and tested the accuracy of this procedure with regard to hemorrhagic and ischemic strokes of varied size and affecting diverse brain regions. To this end, we used CT images from 24 patients with acute stroke, as well as the same 72 control subjects used for the analysis of the simulated lesions. The patients were specifically selected to create four groups of equal numbers, and they were characterized by the presence of either a hemorrhagic or ischemic stroke and of either focal or extended lesions (see Table 1). A trained operator with several years' experience performed a manual delineation of the lesions in the stroke CT images, using MRIcron (McCausland Center for Brain Imaging, Columbia, SC, USA). The operator drew each lesion on a ‘slice-by-slice’ basis, using an axial view of the CT image. In practice, the area deemed to be lesioned was outlined using a mouse, and then a filling function was used to incorporate all the brain mass lying within those borders into a lesion map. The manually delineated lesion was employed as a reference to quantify the performance of the automated method in terms of DSI (Eq. 2). To complement the analysis of stroke CT images, we also measured the sensitivity and the positive predictive value associated with the automated lesion detection. After calculating the number of true positives (TP), false positives (FP), true negatives (TN) and false negatives (FN), we quantified the detection sensitivity as TP / (TP + FN) and the positive predictive value as TP / (TP + FP). Under this formulation, the DSI can be expressed as 2 × TP / [(FP + TP) + (TP + FN)].

In addition, we applied the automated lesion delineation procedure to all stroke CT images collected for the BUCS project, and used the resulting lesion maps for a voxel-based lesion symptom mapping (VLSM) analysis (Bates et al., 2003) to provide an example of the potential utility of our method in neuropsychology. This was carried out in relation to the symptoms of unilateral neglect in patients. We selected a global index of the severity of unilateral neglect for each patient (Humphreys et al., 2011) and used available behavioral data from 332 patients (161 females, 73 ± 12 years old) to perform VLSM. CT images for which no behavioral data were available were excluded from this analysis; we therefore were able to analyze 332 brain lesions with VLSM. A linear model was fit at each voxel, relating the neglect score to lesion intensity (0 for no lesion; 1 for lesion). Tests were confined to those voxels for which there were at least ten patients with and ten patients without a lesion. A statistical threshold cut-off (*t*-value) was determined based on permutation testing (*n* = 1000) with a significance level of 0.01 ((Kimberg et al. 2007)). Specifically, we randomly reassigned the patients' behavioral scores 1000 times, and for each permutated dataset, we refit the GLM and recorded the size of the largest *t*-values. Based on those values, we created a null distribution against which the significance of the actual *t*-scores obtained by VLSM was then assessed.

## 3 Results

### 3.1 Preprocessing of CT images

**Fig. 3:**
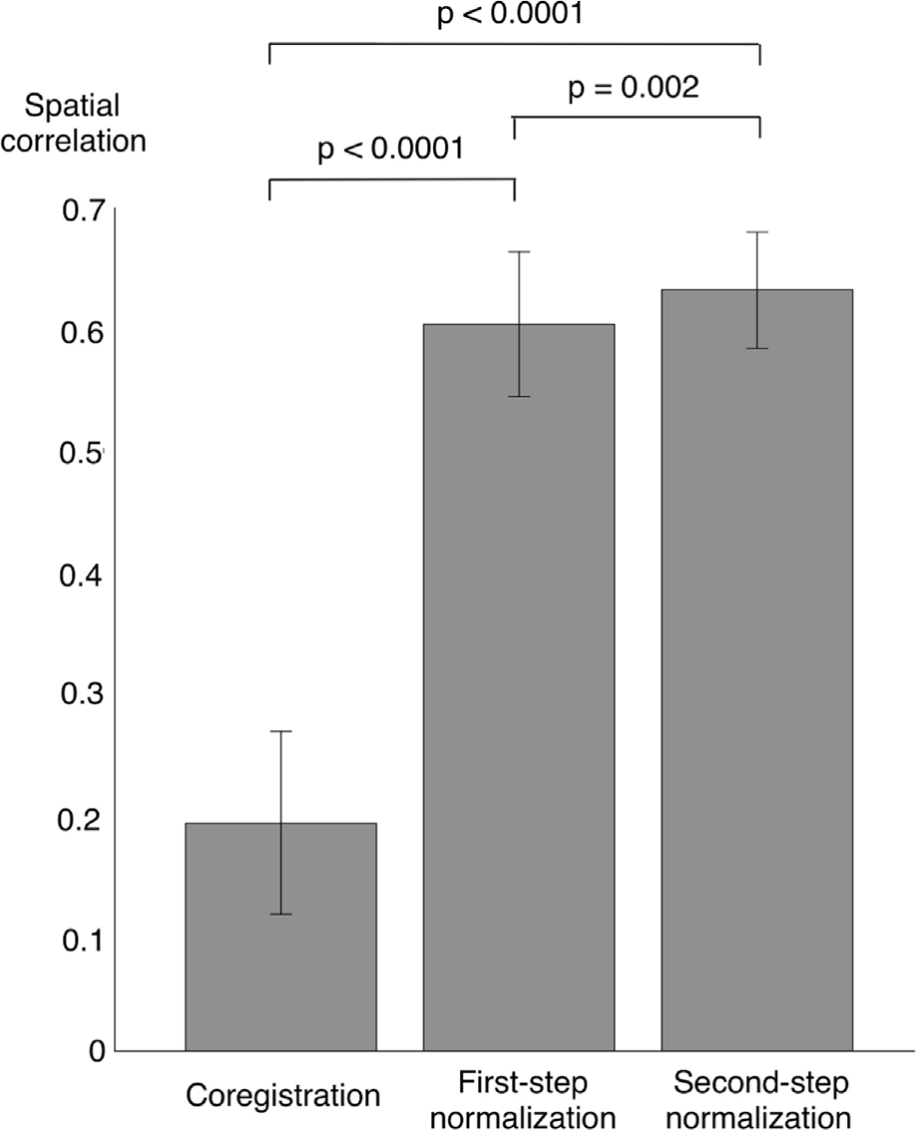
Spatial normalization of CT images: quantitative analysis.Using spatial correlation, we measured the correspondence between the CT image at different preprocessing steps (coregistration, first-step normalization, second-step normalization) and the CT template. This analysis was performed on a group of 72 control CT images. Average and standard deviation values are reported for each processing step. Significant differences between correlation values of processing steps, calculated using paired *t*-tests, are indicated in the figure.

One important feature of our lesion mapping method is the precise spatial registration of each CT image to a common space, so that a voxel-by-voxel statistical comparison could be performed of a single stroke scan against a group of normal scans. Accordingly, we evaluated the effectiveness of this spatial registration, and specifically quantified the improvement in spatial registration across the different image processing steps (coregistration, first-step normalization, second-step-normalization). The analysis of spatial correlations with the CT template revealed that each of these steps significantly improved the spatial registration, resulting in significant increases in moving from coregistration to first-step normalization and from first-step normalization to second-step normalization (Fig. 3).

We visually inspected the normalized images, from control CT scans and from stroke CT scans, ensuring that the registration to a common space proceeded correctly in all cases (see Supplementary Fig. 3 for some examples). The normalized images from control CT scans were processed to create an average map and a standard-deviation map (Supplementary Fig. 4), which were then used to define normal intensity ranges for lesion mapping. Notably, the average CT map largely corresponded to the template image. However, the standard deviation map contained relatively higher values near the brain contours and the ventricles. This indirectly indicates that inter-subject differences in brain shape were not completely eliminated.

**Fig. 4:**
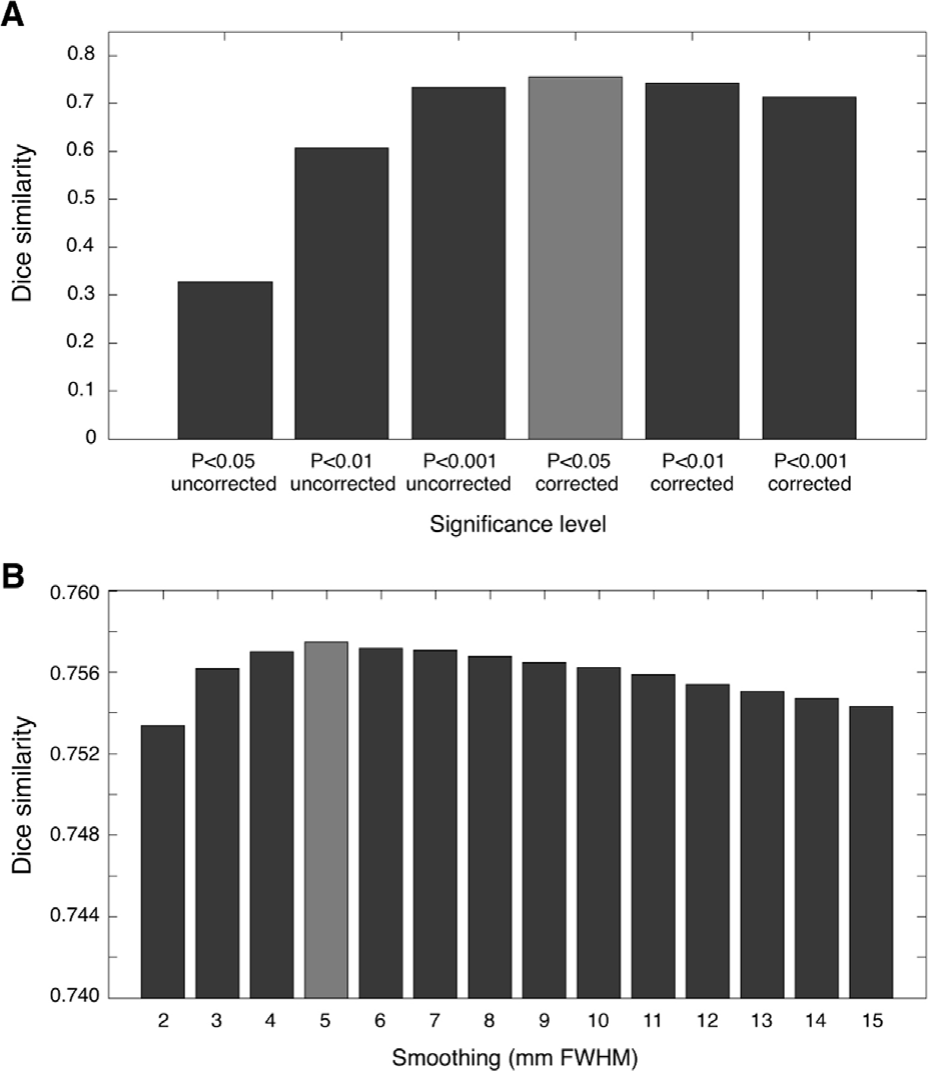
Lesion detection on simulated data: significance thresholding and image smoothing.We assessed the correspondence between the lesion masks used to generate the simulated CT images (ground truth) and those obtained by the automated lesion detection method on the same images. (A) We tested six different significance levels: *p* < 0.05, *p* < 0.01 and *p* < 0.001, with and without correction for multiple comparisons. Among them, the significance thresholding at *p* < 0.05 corrected (indicated with a light gray bar) was selected. (B) After selecting the significance level, we examined the results for smoothing values ranging between 2 and 15 mm full-width half maximum (FWHM). The selected configuration was 5 mm FWHM (also indicated with a light gray bar).

### 3.2 Detection of simulated lesions

By comparing the simulated and detected lesion images, we quantified the performance of the procedure under controlled conditions. Here, the DSI was used as a measure of overall spatial overlap between simulated and the detected lesions. We started our investigation on the simulated data by examining the dependence of the lesion detection results on the degree of smoothing and the level of significance thresholding. Values producing the highest DSI indicated the specific parameters i.e. 5-mm FWHM smoothing and *p* < 0.05 Bonferroni-corrected thresholding needed to optimize automated lesion detection (Fig. 4A–B).

**Fig. 5:**
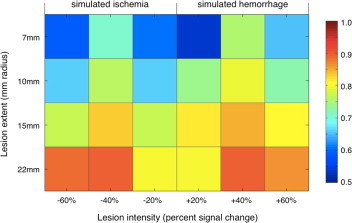
Automated lesion detection on simulated data: effects of lesion size and contrast. We assessed the DSI between the lesion masks used to generate the simulated CT images (ground truth) and those obtained by the automated lesion detection method using optimal significance thresholding (*p* < 0.05 corrected) and image smoothing (5 mm FWHM). Specifically, we examined detection performance for spherical lesions of 7, 10, 15 and 22 mm radii and intensity increases/decreases of 20%, 40% and 60% compared to the mean image intensity. Intensity increases and decreases simulated hemorrhagic and ischemic strokes, respectively.

We then selected these parameters for further analyses, and investigated the performance of the procedure on simulated lesions of different sizes and relative intensities. We observed that all configurations were characterized by reasonably accurate lesion identifications, with DSI ranging between 0.52 and 0.89 (Fig. 5). Nevertheless, the automated method was relatively less sensitive in the presence of lesions that were small in size and/or were characterized by low image contrast (i.e. ±20% deviation from normal intensity values). Finally, simulated hemorrhagic and ischemic strokes yielded very similar average DSI (0.68 and 0.67, respectively).

### 3.3 Detection of actual lesions

**Fig. 6:**
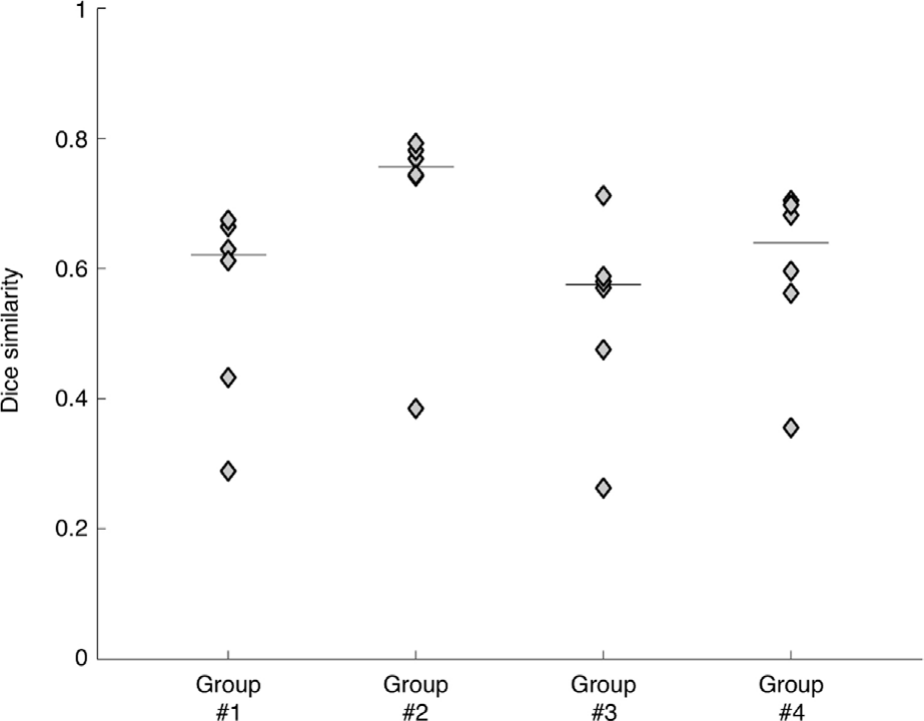
Lesion detection accuracy on stroke CT scans.We measured the DSI between brain lesions obtained from automated and manual classifications. The 24 cases are divided into four groups of equal number: group 1, focal hemorrhagic; group 2, extended hemorrhagic; group 3, focal ischemic; and group 4, extended ischemic. Each individual case is indicated with a diamond marker. The median value across the six elements of each group is indicated with a horizontal line.

We started our analysis of actual CT images by assessing the potential for false positives, using settings determined with the simulated lesion data (smoothing: 5 mm FWHM; significance thresholding: *p* < 0.05 Bonferroni corrected). We analyzed the five control scans used in the creation of simulated lesions (see Methods section), as these were patient scans with no visible lesions. When we applied our automated lesion delineation procedure, we found that only 0.013% and 0.145% of the total number of brain voxels showed significantly positive and negative variations, respectively. Across the five control images used for the analysis, the number of voxels detected as belonging to a lesion ranged from 1301 to 3714, against a total number of about 1.6 million voxels in the brain. Subsequently we proceeded to the analysis of the 24 stroke CTs, comparing the results of the manual and automated lesion classifications. The automated procedure was able to recover lesion areas in all cases (see Fig. 6). We observed a substantial variability in the DSI for CT scans within a given stroke group (hemorrhagic and ischemic, focal and extended strokes). An analysis of the median values across these four groups revealed that the performance of the method was generally better for large, rather than small lesions, and for hemorrhage rather than ischemia (Fig. 6). Complementary analyses suggested that higher DSI in hemorrhagic than in ischemic cases was the consequence of a lower number of false negatives (i.e. greater sensitivity) and improved performance for larger lesions was associated with a lower number of false positives compared to true positives (i.e. greater positive predictive value) (Supplementary Fig. 5).

**Fig. 7:**
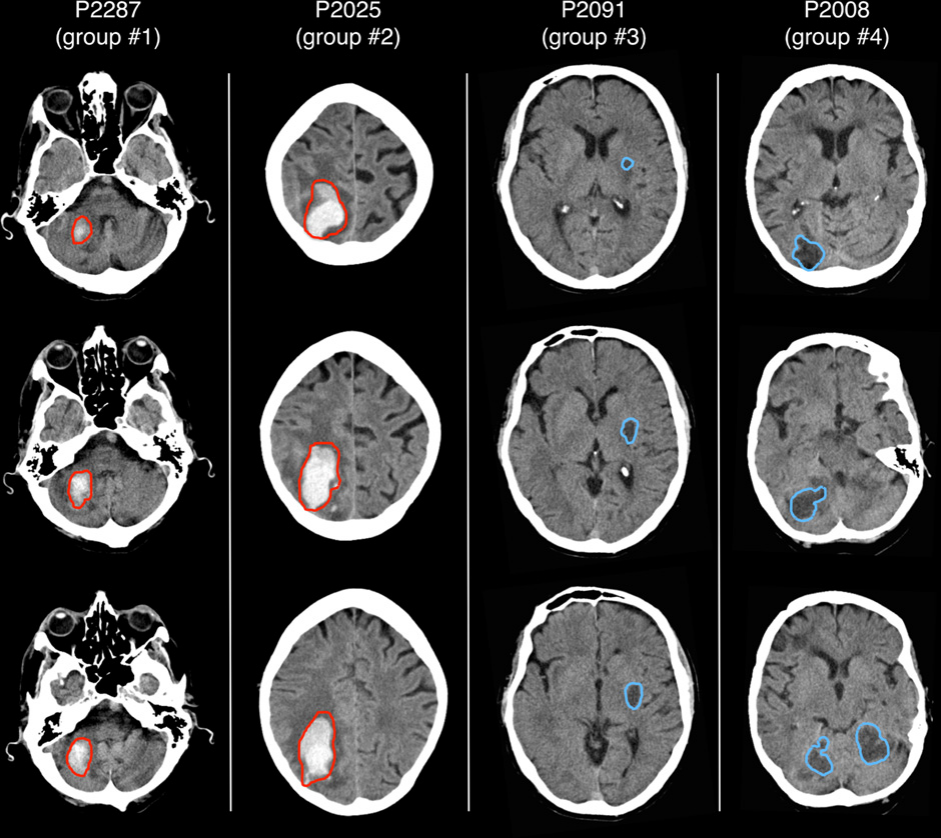
Automated detection of brain lesions from stroke CT scans.In this figure we show brain lesions obtained by the automated method on four different cases, each belonging to a different group: group 1, focal hemorrhagic; group 2, extended hemorrhagic; group 3, focal ischemic; and group 4, extended ischemic. Red and blue outlines define the lesion area and correspond to significant positive and negative increases, respectively, compared to the control group. A minimum cluster size of 1400 voxels (1.4 cm^3^) was applied to the lesion maps for visualization purposes.

**Fig. 8:**
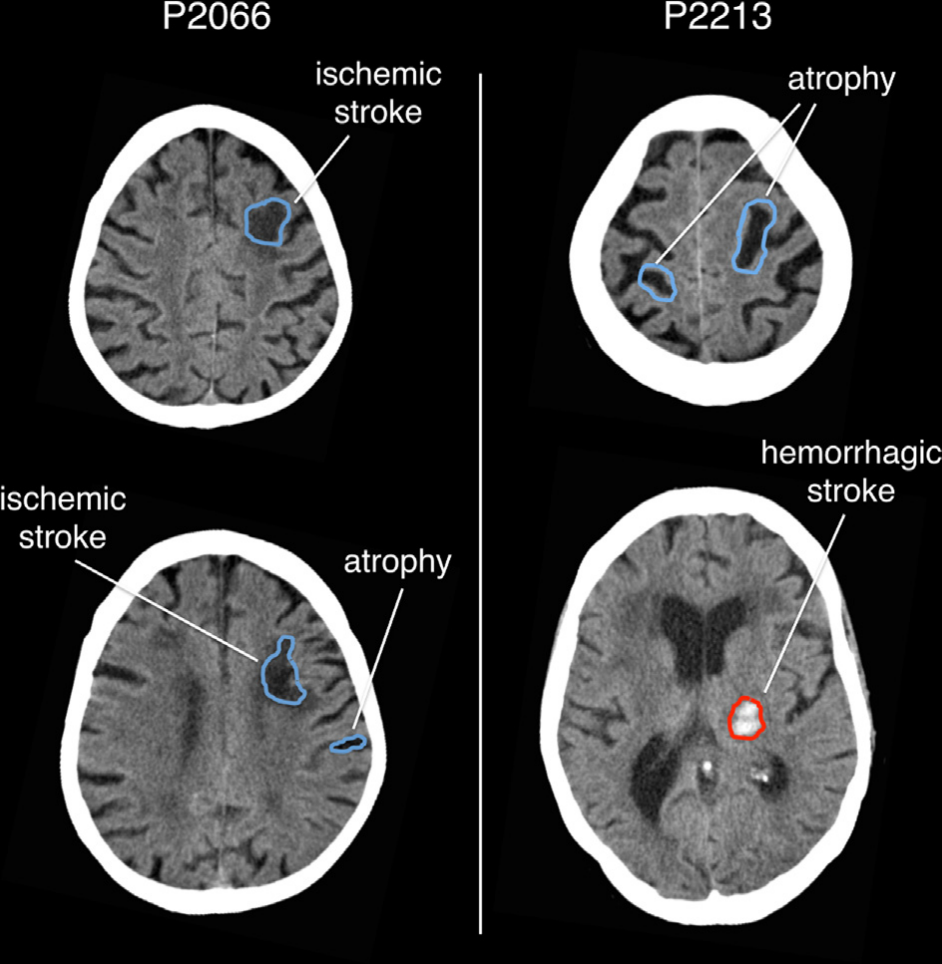
Detection of stroke and atrophy on stroke CT scans. In this figure we show two cases for which the automated method retrieved lesion areas that are ascribed not only to a hemorrhagic or ischemic stroke, but also to brain atrophy. Red and blue outlines define the lesion area and correspond to significant positive and negative increases, respectively, compared to the control group. A minimum cluster size of 1400 voxels (1.4 cm^3^) was applied to the lesion maps for visualization purposes.

Visual inspection of the binary maps generated by the automated method, performed using a cross-hair cursor in MRIcron, confirmed that the central region of the stroke-induced lesion was always retrieved. We found that lesion-boundary reconstructions were precise in 18 out of 24 cases (see Fig. 7). When we inspected cases having relatively low DSI between automated and manual lesion delineations, we observed that the automated method indicated additional areas not classified by the manual operator as stroke-related. These areas were generally characterized by reduced image intensity, which may be attributed to the presence of brain atrophy (Fig. 8).

**Fig. 9:**
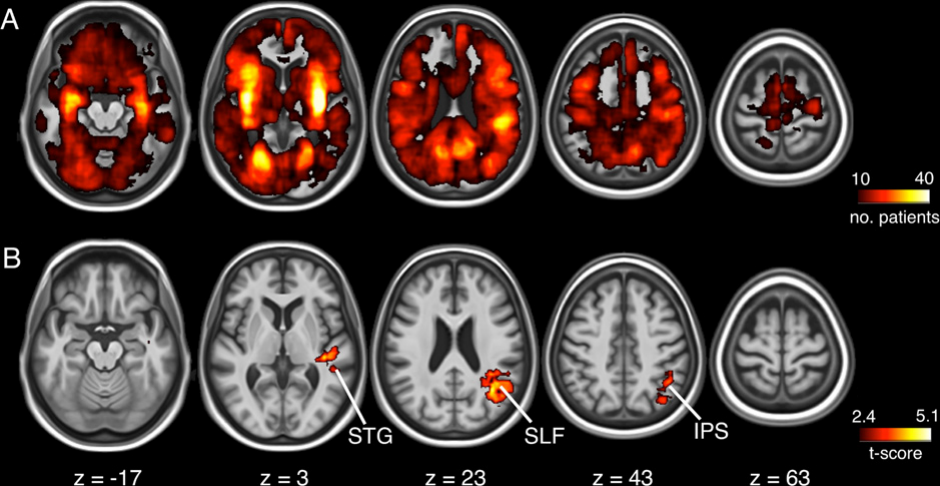
Lesion correlates of visuospatial neglect revealed by VLSM. (A) Lesion overlap map for the 332 patients used in the VLSM analysis. (B) VLSM indicated visuospatial neglect to be associated with lesions in right intraparietal sulcus (IPS), right superior longitudinal fasciculus (SLF) and right superior temporal gyrus (STG). Only significant voxels are shown based on a critical threshold determined by permutation testing (*p* < 0.01).

Finally, we evaluated the usefulness of automatically delineated lesions for large-scale studies of brain–behavior relationships. To this end, we used neglect severity indices estimated by neuropsychological testing in 332 patients and their corresponding CT lesion maps. The spatial distribution of the lesions across patients was very variable, such that any brain voxel was lesioned in maximum 40 patients, and almost the whole brain was lesioned in at least 10 patients (see Fig. 9A). VLSM analysis yielded regions exclusively in the right temporo-parietal cortex, with three main clusters in the intraparietal sulcus, superior longitudinal fasciculus and superior temporal gyrus, respectively (Fig. 9B). This result is in line with previous literature, which implicated such regions in visuospatial neglect.

## 4 Discussion

In this study, we have presented a novel method for the automated delineation and classification of stroke lesions from brain CT images and have shown its effectiveness for both simulated and real stroke lesions. Significantly, this automated method can reliably detect brain lesions with various characteristics, both in terms of size and with regard to signal hyper- or hypo-intensity. It has the potential for wide application, including aiding in the creation of large patient lesion databases for use in neuropsychological research (see Fig. 9 for an example) or for quantitative lesion analyses which may facilitate future neurorehabilitation protocols.

Our method has been specifically developed for the analysis of stroke CT images. It is based on the normalization of the CT scan to a common space and subsequent comparison to a normative CT sample for the detection of outlier regions (Stamatakis and Tyler, 2005). The method provides an alternative to current techniques including those that rely on rule-based expert systems for labeling background, bone, gray and white matter, cerebrospinal fluid, and stroke lesions (Matesin et al., 2001) and those that use unsupervised learning methods based on image histograms and compare the CT images of the two hemispheres in order to segment stroke regions (Usinskas et al., 2004). It must be pointed out that almost all previously proposed approaches were dedicated to the exclusive detection of either hemorrhagic or ischemic regions. Only one method has been previously proposed to deal with both hemorrhagic and ischemic strokes in CT scans (Chawla et al., 2009). This earlier method compared image intensities between the two hemispheres, and therefore could not detect bilateral lesions. In contrast, our method does not have such a limitation, but classifies hemorrhage and ischemia on the basis of significant signal hyper- and hypo-intensities, defined in a voxel-by-voxel manner with respect to a normative sample.

One of the most important features of our automated method is the accurate normalization of the CT image to MNI space, which is achieved in two successive warping steps (Fig. 1) and uses a recently-published digital template (Rorden et al., 2012). It is noteworthy that our normalization procedure does not require the segmentation of gray and white matter voxels, as commonly done in several MR-based lesion delineation approaches in order to improve spatial registration (Seghier et al., 2008). We opted not to include a segmentation step in our processing pipeline, as the image contrast in the CT image is relatively low, and this procedure can impair segmentation accuracy (Rekik et al., 2012). Furthermore, we could not simply rely on classical MR-based methods for spatial normalization, as these typically fail when applied to CT scans (Rorden et al., 2012). To address this problem, we developed a normalization approach tailored to low-contrast images, and that incorporated information on brain contour and ventricle masks, yielding improved registration to template space. Moreover, spatial registration guided by brain contour and ventricle masks significantly reduced the difficulties associated with spatial normalization of stroke CT scans, for two main reasons. First, hyper- or hypo-intensities in stroke CT scans can confound or bias normalization, usually yielding an under- or over-fitting of the lesioned region, respectively (Ripolles et al., 2012). This may explain the fact that we found higher DSI with simulated lesions having an intermediate lesion intensity change (Fig. 5). Secondly, taking into account the overall brain morphology helps optimize image normalization of stroke CT scans because the infarct may distort adjacent tissues e.g. by causing midline shifts or constricting the ventricles (Rekik et al., 2012).

Our analysis also revealed the limited impact image smoothing has on the accuracy of lesion delineation using our automated method (Fig. 5B). Although this result is seemingly at odds with previous MR studies (Stamatakis and Tyler, 2005), it may be explained by the fact that CT scans typically have lower spatial resolution and are more blurred than MR scans. Accordingly, the effects of smoothing are not apparent at the level of the single image. The use of spatial smoothing is nonetheless justified by the necessity of accounting for inter-subject anatomical variability and to satisfy the assumption of normality for parametric tests. Consistent with MR studies, smoothing values with a kernel that is comparable to the minimum lesion size appeared to ensure optimal performance of the automated method (Stamatakis and Tyler, 2005). Larger smoothing values are presumed to penalize the detection of small lesions, as these tend to become blended with the background signal surrounding the lesion itself. It is also worth noting that smoothing may have an impact on the precise delineation of lesion boundaries (Seghier et al., 2008). Indeed, strokes do not present as a uniformly reduced signal with respect to normal tissue, but display smaller decreases on the borders and larger decreases towards the center of the lesion (Rekik et al., 2012). This implies that our method is generally less accurate at the borders than at the center of the lesion.

Our automated method implements a statistical mapping approach based on the Crawford–Howell *t*-test for case–control comparisons, a robust and widely used approach in other research areas, such as neuropsychology (Crawford et al., 2009; Crawford and Howell, 1998). Critically, such comparisons between images of brain-damaged and healthy subjects assume that differences relate only to the presence or absence of abnormal tissue. Thus, it is important to minimize potential differences arising from demographics, particularly age, by selecting appropriately-matched control CT scans or by factoring out these variables within the statistical framework (Crawford et al., 2011). For instance, the comparison of an elderly stroke patient to younger control subjects might detect abnormal voxels representing atrophy rather than a real injury produced by stroke (Earnest et al., 1979). The degree of abnormality is expressed in terms of *t*-scores, hence provides quantification on a continuous scale. Furthermore, based on the *t*-score maps, we delineated damaged areas by binarization, using a preset significance level to define a cut-off value: regions with *t*-scores larger than a positive threshold value were classified as resulting from hemorrhage; conversely, those with *t*-scores smaller than a negative threshold value were classified as caused by ischemia. The resulting lesion map, generated within stereotaxic space, can easily be compared with a given brain atlas for lesion overlap and lesion–symptom mapping analyses (Bates et al., 2003).

During the evaluation of our method on actual lesions, we used manual classification as the reference for calculating the DSI. The automated lesion detection method generated lesion maps that were always spatially consistent with the manual detection in terms of lesion location, and in many cases the correspondence of automated and manual lesion maps was striking (Fig. 6). On stroke CT scans, we found that larger lesions were delineated more accurately than small lesions. This may be not only an effect of smoothing, as discussed above, but also a consequence of a different surface-to-volume ratio in larger lesions. Furthermore, the detection of hemorrhagic stroke lesions was generally more reliable than that for ischemic strokes. This may simply be due to the fact that lesion boundaries in the CT images are typically better defined for hemorrhagic than ischemic strokes. However, there may also be alternative explanations. We observed that the analysis of CT images from participants without stroke yielded about ten times more voxels with significantly reduced (0.145%) than with significantly increased intensities (0.013%). It should be considered that the detection of areas with hypo-intense signals in the CT scans might not only be the result of ischemic stroke lesions but could also represent brain atrophy (Fjell et al., 2009; Yamaura et al., 1980). Consistent with this possibility, we found that in some cases the automated method detected brain regions that were not manually delineated, and that were compatible with brain atrophy (Fig. 8). It is important to consider that brain atrophy, especially in the chronic phase, may be a consequence of diaschisis induced by the lesion itself (Price et al., 2001). In the first instance, the inability to discriminate between ischemic stroke and atrophy may be considered a limitation of our method. On the other hand, it might also be important to consider not only brain lesions but also brain atrophy in attempts to account for behavioral deficits in terms of lesion–symptom maps or prediction, as the atrophy could contribute to the behavioral symptoms.

With regard to the lesions resulting from stroke, we noticed that boundaries did not completely correspond to those obtained by manual delineation (Fig. 7). This may be ascribed to the methodological limitations of spatial normalization, smoothing and statistical mapping discussed above. It should be noted that the precise definition of lesion boundaries on stroke CTs is not straightforward, even with the manual approach (van der Worp et al., 2001), due to the limited spatial resolution and contrast of CT images. In future research, we hope to further develop our method so as to improve the degree of correspondence with manually-delineated lesions. Possible solutions could involve the detection of adaptive smoothing approaches to better preserve lesion features in stroke CT scans (Tsai et al., 2005) and the use of Bayesian statistics to control for the effect of demographic variables in comparisons between single patients and controls (Crawford et al., 2011).

## 5 Conclusion

We have validated a fully-automated method for lesion detection and classification in CT stroke scans. This relies on the spatial alignment to the CT image to template space and the detection of voxel outliers based on a group of control CT images. Manual tracing methods remain the standard technique for delineating damaged brain regions; nevertheless our approach has important advantages: it is fully automated, produces lesion images in a common MNI space which is useful for lesion–symptom mapping, can deliver significant time savings and eliminates inter-operator differences that are intrinsic to the manual approach. These benefits may prove critical for the development of novel applications, such as the creation of large-scale lesion databases for use in neuropsychological research. Our method can also provide quantitative information about lesion extent and location, which may be used to trace longitudinal changes in damaged tissue in a reproducible and objective manner.

## Appendix A Supplementary Materials

Supplementary for Automated delineation of stroke lesions using brain CT images.
