# Supplementary material for: Automated delineation of stroke lesions using brain CT images
Source: Neuroimage Clin. 2014 Mar 21;4:540–8. doi: 10.1016/j.nicl.2014.03.009 (PMC3984449; doi:10.1016/j.nicl.2014.03.009)
Supplement: Supplementary file 1 — Supplementary for Automated delineation of stroke lesions using brain CT images. [file mmc1.docx]

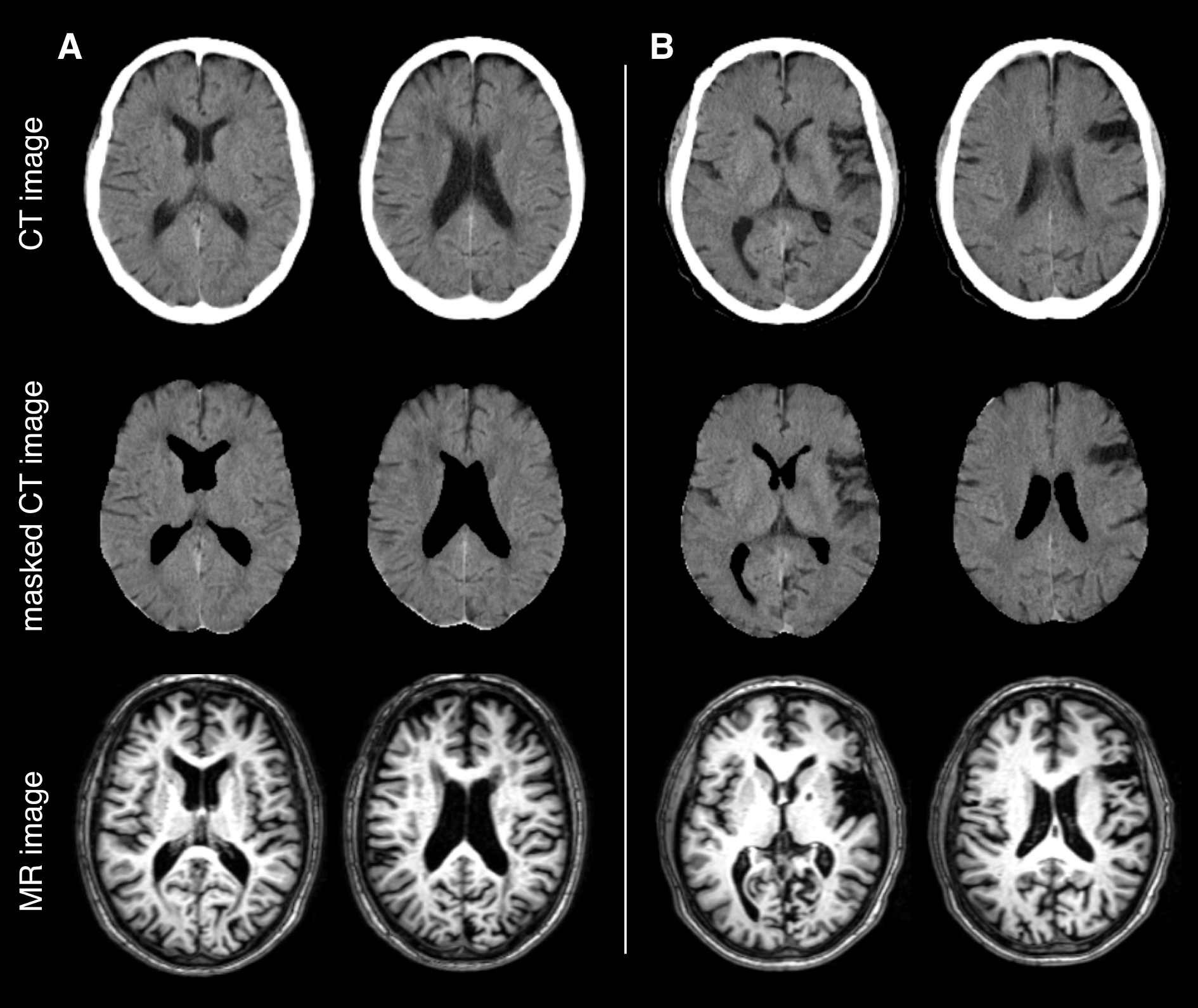


**Supplementary Figure 1. CT data processing: definition of ventricle and brain masks.** We implemented a thresholding approach based on the intensity histogram to reconstruct ventricle and brain contours. In this figure we show two selected axial sections of images from a control subject (A) and a stroke patient (B). For each of them, the CT image before and after masking of ventricle and brain contours, as well as an MR image of the same subject, are compared.


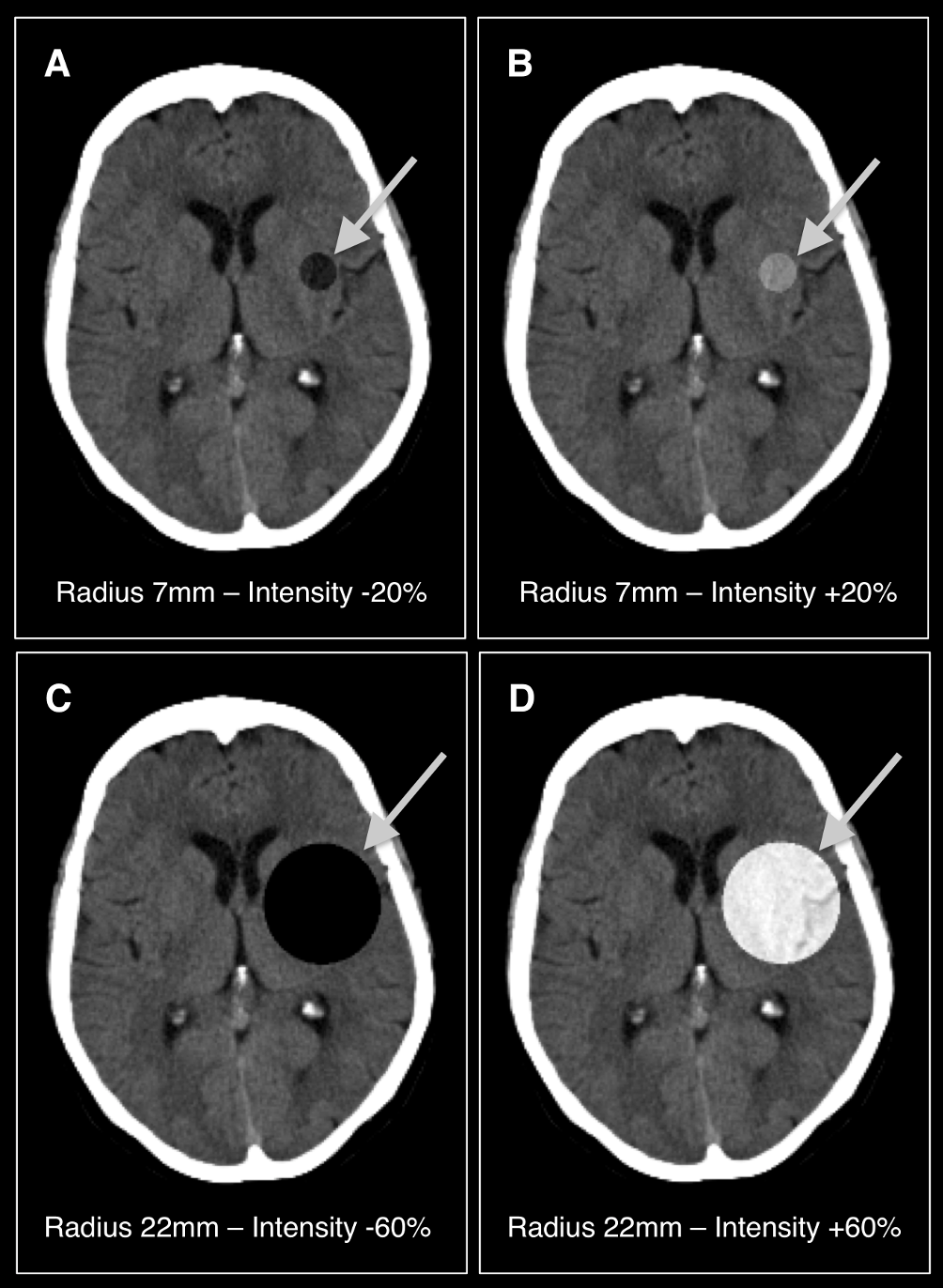


**Supplementary Figure 2. Examples of simulated lesions in CT images.** We generated spherical masks with radius of 7, 10, 15 and 22 mm. These masks were superimposed onto control CTs (where no lesion was present) with relative intensity equal to -60%, -40%, -20%, +20%, +40%, +60% of the average image intensity. This figure shows examples of simulated lesions with 7mm radius and -20% relative intensity (A), 7mm radius and +20% relative intensity (B), 22mm radius and -60% relative intensity (C), 22mm radius and +60% relative intensity (D).

**
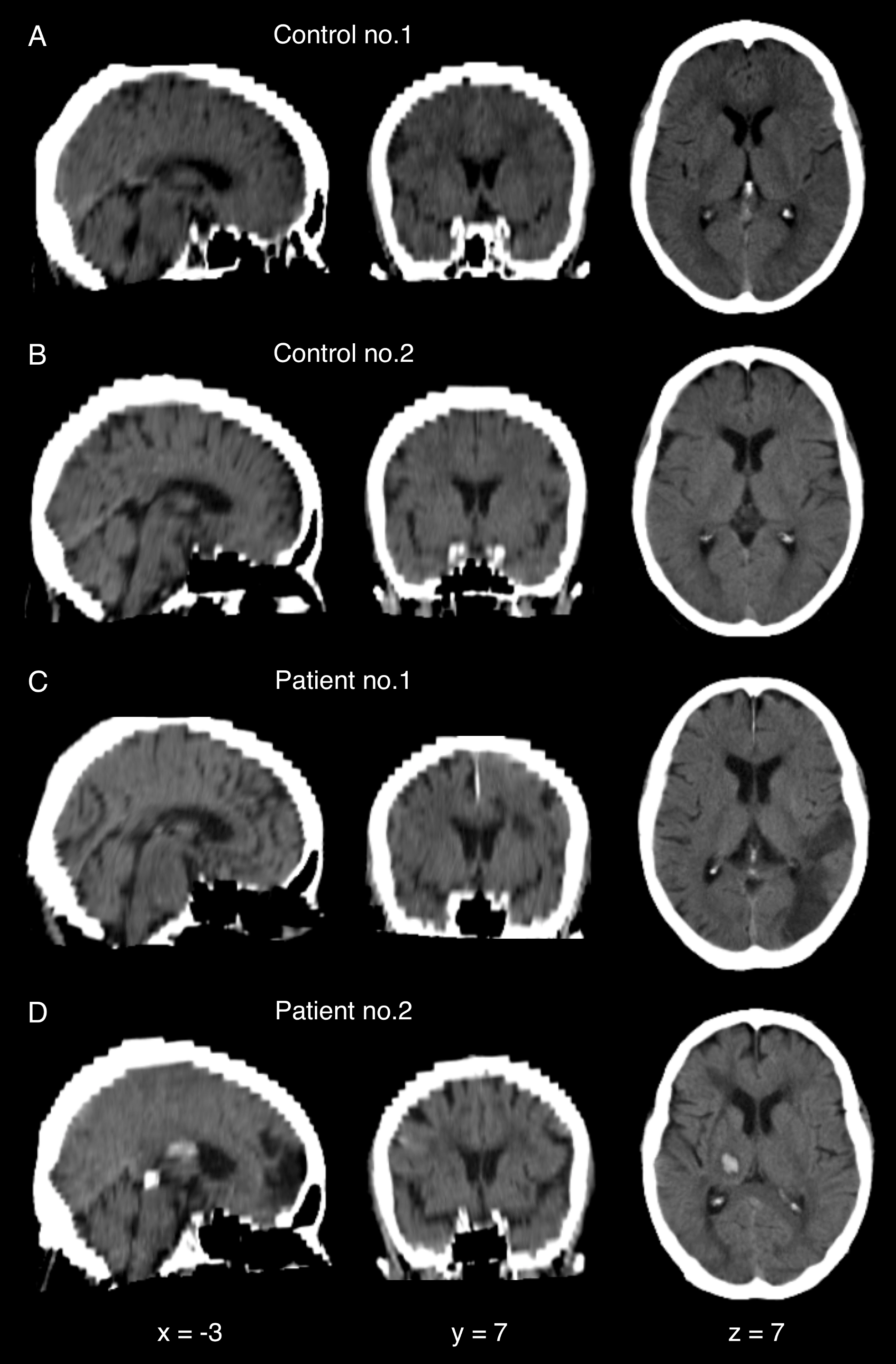
**

**Supplementary Figure 3. Examples of CT images in MNI space.** In this figure we show the CT images of two representative subjects, two controls (A-B) and two stroke patients (C-D), after transformation to MNI space. Images are represented in sagittal, coronal and axial sections.


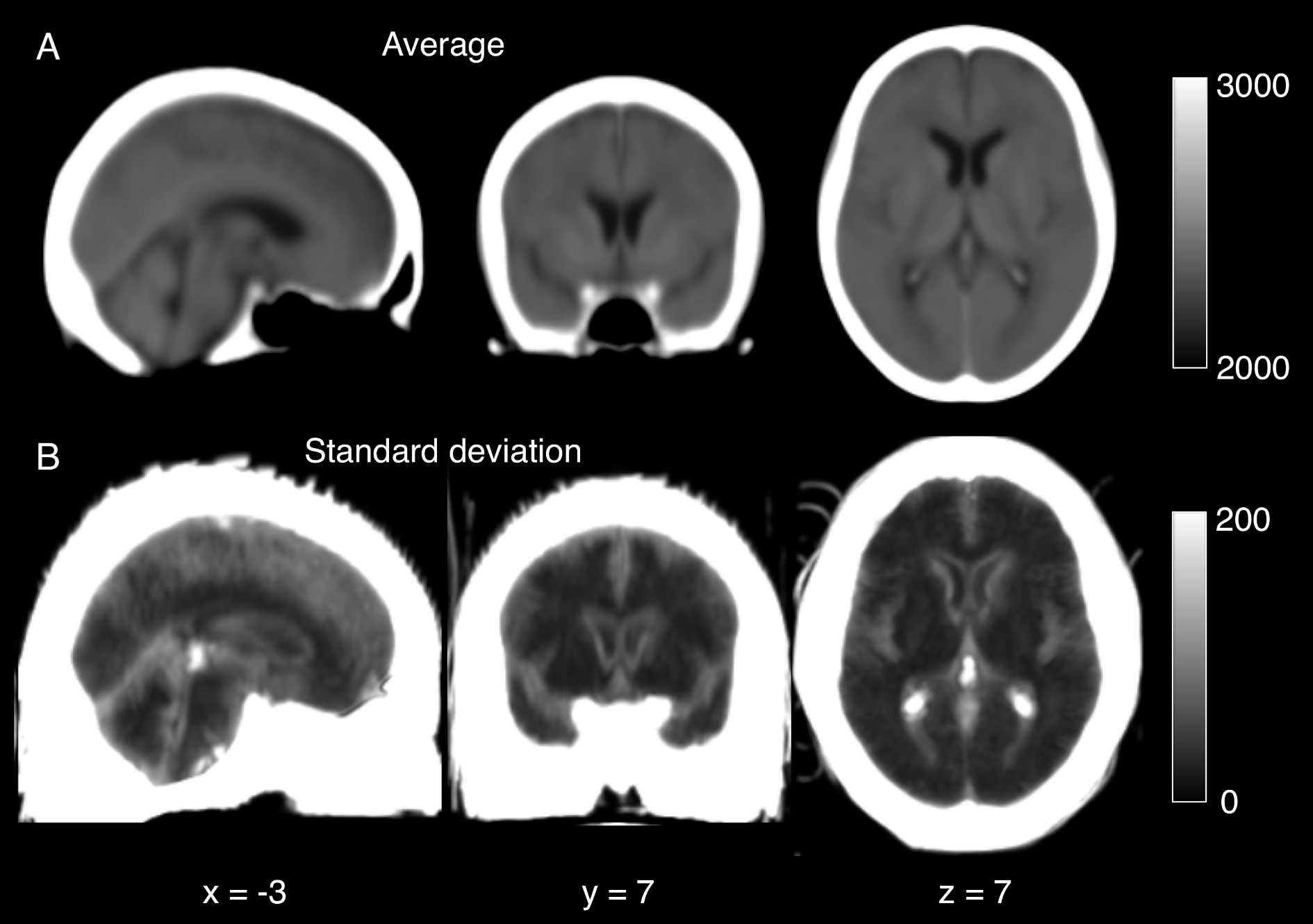


**Supplementary Figure 4. Creation of average and standard deviation images from control CT scans.** Using the whole set of control CTs in MNI space, an average map (A) and standard deviation map (B) were generated. These maps were used to define a range of normal values to classify image intensities in stroke CTs.

**
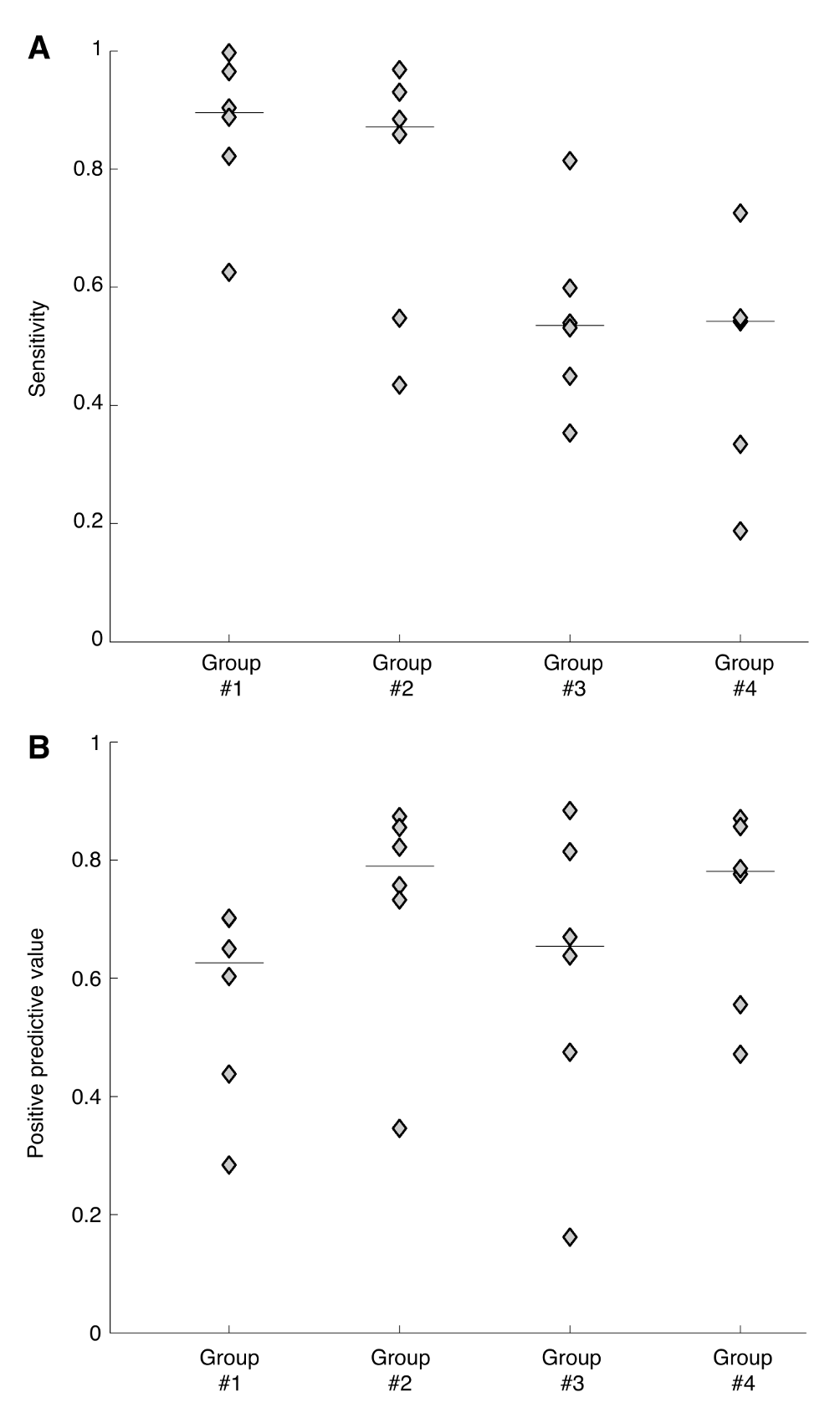
**

**Supplementary Figure 5. Automated lesion detection: sensitivity and positive predictive value.** We quantified (A) sensitivity and (B) positive predictive value of automated lesion detection, using the manually delineated lesion as reference. The 24 cases are divided in four groups of equal number: group #1, focal hemorrhagic; group #2, extended hemorrhagic, group #3, focal ischemic; group #4, extended ischemic. Each single case is indicated with a diamond marker. The median value across the six elements of each group is indicated with a horizontal line.
